# Psychotherapies for depressive disorders: evidence synthesis with a focus on schema therapy and third-wave approaches

**DOI:** 10.1192/j.eurpsy.2026.11445

**Published:** 2026-07-31

**Authors:** M. S. Artemieva, A. Medvedev, C. Aleksashina, A. Shadrikova

**Affiliations:** 1 Peoples’ Friendship University of Russia named after Patrice Lumumba; 2 Psychiatric Clinical Hospital No. 4 of the Moscow Health Department, Moscow, Russian Federation

## Abstract

**Introduction:**

Depressive disorders remain a major public-health burden. Beyond pharmacotherapy, outcomes are limited by adherence problems, cognitive rigidity, and psychosocial stressors, sustaining demand for effective, evidence-based psychotherapies. Retail antidepressant sales rose markedly in 2020–2021 vs 2019, underscoring the scale of need.

**Objectives:**

To summarise evidence-based psychotherapies for depressive disorders and appraise the emerging role of schema therapy.

**Methods:**

Narrative review of publications retrieved from eLibrary, CyberLeninka, and PubMed using predefined keywords on non-pharmacological treatment of depression; 78 records identified, 35 cited.

**Results:**

Cognitive-behavioural therapy (CBT) and interpersonal psychotherapy (IPT) have the strongest evidence (Level I), supported by reviews/meta-analyses; CBT targets cognitive distortions with behavioural activation and skills training, while IPT addresses role transitions, grief, and interpersonal conflict. Third-wave approaches show favourable data: Acceptance & Commitment Therapy (ACT) and mindfulness-based CBT (MBCT) reduce depressive symptoms via cognitive defusion/acceptance, with meta-analytic support. Short-term psychodynamic psychotherapy (STPP) holds Level II evidence; intensive short-term dynamic psychotherapy (ISTDP) is effective for mood disorders and may be cost-effective. Group formats based on CBT/IPT also carry Level I support.

Schema therapy (ST) integrates cognitive-behavioural, experiential, and psychodynamic/object-relations elements and targets early maladaptive schemas/modes—mechanisms central to chronic depression. Evidence is growing: reviews and clinical studies note efficacy, often requiring longer courses; case-series and pragmatic data report stable remission after ~60 sessions and 68% improvement in a 100-patient sample.

**Conclusions:**

Across psychotherapies for depression, CBT/IPT (including group formats) show Level I evidence; STPP/ISTDP have supportive Level II data; ACT/MBCT are promising with meta-analytic support. Schema therapy is a viable option—particularly for chronic and personality-comorbid depression—though typically requiring longer treatment courses; service pathways should incorporate ST alongside CBT/IPT to address schema-level and interpersonal maintaining mechanisms.

**Disclosure of Interest:**

None Declared

